# The formation of higher alcohols in rice wine fermentation using different rice cultivars

**DOI:** 10.3389/fmicb.2022.978323

**Published:** 2022-10-28

**Authors:** Chunxiao Wang, Guoyi Yuan, Yulin He, Jiadai Tang, Hongxiang Zhou, Shuyi Qiu

**Affiliations:** ^1^Province Key Laboratory of Fermentation Engineering and Biopharmacy, Key Laboratory of Plant Resource Conservation and Germplasm Innovation in Mountainous Region (Ministry of Education), School of Liquor and Food Engineering, Guizhou University, Guiyang, China; ^2^Guizhou Maotai-flavored Liquor Group Production Co., Ltd., Guiyang, China; ^3^Department of Liquor Engineering, Moutai Institute, Renhuai, China

**Keywords:** glucose, correlation analysis, *S. cerevisiae*, higher alcohol, nutrient consumption

## Abstract

Higher alcohols are closely related to the flavor and safety of rice wine. The formation of n-propanol, isobutanol, isoamyl alcohol, and phenylethanol during rice wine fermentations was for the first time investigated in this study among 10 rice cultivars from two main production regions. Rice wine made from Yashui rice, the long-grain non-glutinous rice from Guizhou, produced the highest yields of higher alcohols (487.45 mg/L), and rice wine made from five glutinous rice cultivars produced the lowest yields of higher alcohols (327.45–344.16 mg/L). An extremely strong correlation was found between the starch in rice and higher alcohols in rice wine. Further analysis first showed that the former fermentation period was key for the nutrient consumption and higher alcohol formation, with more than 55% of glucose being consumed and more than 75% of higher alcohols being synthesized in 48 h. Correlation analysis confirmed the strong correlation between nutrient consumption and higher alcohol formation including valine–isobutanol (coefficient higher than 0.8 in seven rice cultivars and higher than 0.6 in three rice cultivars), glucose–isoamyl alcohol (coefficient higher than 0.8 in five rice cultivars and higher than 0.6 in the other five rice cultivars), and glucose–phenylethanol (coefficient higher than 0.8). The correlation of threonine–n-propanol, leucine–isoamyl alcohol, phenylalanine–phenylethanol, glucose–n-propanol, and glucose–isobutanol varied among the rice wines made from 10 rice cultivars. RT-qPCR analysis on five target genes verified the variation caused by different rice cultivars. this study for the first time reported the special formation pattern of higher alcohols during rice wine fermentation, emphasizing the early contribution of glucose metabolism on the formation of isobutanol. This study highlighted the significance of rice selection for making rice wine with good quality and provided theoretical references for the control of higher alcohols, especially in the former period of rice wine fermentation.

## Introduction

As one of the oldest low-alcohol beverages in the world, rice wine is traditionally fermented from rice (Wei et al., 2016). Chinese rice wine has gained the increased interests from young and female consumers in recent decades with a sales value of US$ 4.3 billion in 2019, which is attributed to its low alcohol, rich nutrients, and healthy effects (Zhang et al., 2015; Wei et al., 2016; Xie et al., 2016). Until now, there have been 17.6 thousand rice wine enterprises in China, which are mainly distributed in South China with Qiandongnan (Guizhou province) ranking the first based on the number of enterprises. South China bounds in rice including red/black/white rice, glutinous/non-glutinous rice, and long-/round-grain rice, which has provided a rich raw material for rice wine production. Recent studies have emphasized the role of rice cultivars in rice wine quality with seven non-glutinous and two glutinous rice cultivars being selected as high-quality materials for making rice wine (Wang et al., 2014; Xie et al., 2016). Therefore, comprehensive studies on the relation of main nutrients in different rice cultivars and the flavor quality of rice wine need to be conducted for elucidating the quality formation of rice wine during fermentation.

Higher alcohols are one of the most important volatile flavor compounds influencing the quality of rice wine (Zhang et al., 2015). A proper amount of higher alcohols in rice wine contributes to its aroma; however, excess higher alcohols (more than 10 g/L) have been reported to be related to hangover and severe headache (Lachenmeier et al., 2008; Li et al., 2017; Sun et al., 2021). Therefore, recent studies have focused on making healthier rice wine by controlling the higher alcohol formation. Fermentative yeasts with low formation ability of higher alcohols were screened or genetically modified, which were mainly *Saccharomyces cerevisiae* (Hirst and Richter, 2016; Li et al., 2018; Wang et al., 2020). A few researchers have also paid attention to the selection of rice cultivars with low production of higher alcohols and the content differences of amino acids among cultivars (Wang et al., 2014; Xie et al., 2016). The formation of higher alcohols has been widely investigated in other alcoholic beverages including wine and beer, which indicated that the starting materials with different contents of carbon and nitrogen greatly affected the formation of higher alcohols (Hirst and Richter, 2016). The metabolism condition in a more complex starting matrix such as various grains was significantly different from that in synthetic media and model grape juice (Hirst and Richter, 2016). As the main nutrients in rice, protein and starch are hydrolyzed into amino acids and glucose by enzymolysis process before rice wine fermentation, providing precursors for the formation of higher alcohols during fermentation. Unlike the liquid-state fermentation of grape juice, the semi-solid-state fermentation of rice wine kept the grain of rice until the end of fermentation, and hence, the complete release of amino acids and glucose from rice was lacking (He et al., 2022), making the rice as a complex starting matrix. The released amino acids are synthesized into higher alcohols mainly by the Ehrlich pathway, but glucose by the Harris pathway. Multigenes have been reported related to the pathways such as *AGP1, GDH1, THR6, BAT1, BAT2, ADH, SFA1*, and *THI3* (Dzialo et al., 2017; Li et al., 2017; Wang et al., 2019, 2021). Therefore, studies on the relation of nutrient consumption by yeast and higher alcohol formation were relevant for controlling the formation of higher alcohols during rice wine fermentation.

Four main higher alcohols have been found in rice wine based on our previous study including isobutanol, isoamyl alcohol, phenylethanol, and n-propanol (Wang et al., 2020). The fermentative yeast with the low formation of higher alcohols has also been screened from Guizhou traditional *Xiaoqu* (Wang et al., 2020). Therefore, the aim of this study was first to further analyze the formation difference of higher alcohols among rice cultivars by the same fermenting *S. cerevisiae* strains. Ten different rice cultivars were selected from two main rice production regions, Guizhou province and the northeast region of China. This study also aimed to correlate the traits of nutrient consumption and the formation of four main higher alcohols, namely, isobutanol, isoamyl alcohol, n-propanol, and phenethyl alcohol, which might provide some references for the raw material selection for rice wine industry.

## Materials and methods

### Rice materials

Ten different rice cultivars were collected from different countries in Guizhou province and the northeast region of China with each coordinate given in Table 1. The main information of the 10 rice cultivars is listed in Table 1 with five rice cultivars being glutinous rice and the other five cultivars being non-glutinous rice. This study used abbreviations of their product names labeling the 10 rice cultivars with BS, YS, TT, WD, PJ, WC, YJ9, YJ7, XHC, and XHY.

**Table 1:** The content of main nutrients in rice and the content of higher alcohols in rice wine.

**Items**	**Rice (cultivar name)**									
	**BS (Baishui)**	**YS (Yashui)**	**TT (Titian)**	**WD (Wandao)**	**PJ (Panjin)**	**WC (Wuchang)**	**YJ9 (Youji)**	**YJ7 (Youji)**	**XHC (Xianghenuo)**	**XHY (Xianghenuo)**
**Basic information of rice**										
Cultivar name	Yuzhenxiang	T-xiangyou-557	Yexiangyou	Yexiangyou	Yanfeng	Jizhan-10	organic*	organic*	Goucendang	Gouyangdang
Rice type	Non-glutinous, long grain, transparent	Non-glutinous, long grain, transparent	Non-glutinous, long grain, transparent	Non-glutinous, long grain, transparent	Non-glutinous, round grain, transparent	Glutinous, round grain, milk white	Glutinous, round grain, milk white	Glutinous, round grain, milk white	Glutinous, round grain, milk white	Glutinous, round grain, milk white
Cultivation region (Coordinate)	Tongren City, Guizhou province (27.73° N, 109.21° E)	Huishui country, Guizhou province (26.13° N, 106.65° E)	Dushan County, Guizhou province (25.69° N, 107.65° E)	Duyun, Guizhou province (26.72° N, 107.53° E)	Panjin, Liaoning province (41.15° N, 122.06° E)	Wuchang country, Heilongjiang province (44.93° N, 127.17° E)	Dalian, Liaoning province (38.92° N, 121.62° E)	Dalian, Liaoning province (38.92° N, 121.62° E)	Congjiang county, Guizhou province (25.58° N, 108.64° E)	Congjiang county, Guizhou province (25.58° N, 108.64° E)
Harvest year	2019	2019	2019	2019	2019	2019	2019	2017	2019	2019
**The content of main nutrients in rice**										
Water (%)	11.91^h^ ± 0.00	13.15^b^ ± 0.00	12.8^c^ ± 0.00	12.07^g^ ± 0.00	12.7^d^ ± 0.00	12.66^d^ ± 0.00	12.3^ef^ ± 0.00	13.28^a^ ± 0.00	12.35^e^ ± 0.00	12.28^f^ ± 0.00
Fat (%)^o^	0.94^a^ ± 0.44	0.57^a^ ± 0.14	1.17^a^ ± 0.13	0.74^a^ ± 0.23	0.65^a^ ± 0.08	0.93^a^ ± 0.35	0.65^a^ ± 0.18	0.42^a^ ± 0.40	1.07^a^ ± 0.09	0.98^a^ ± 0.09
Protein (%)	10.03^a^ ± 0.64	5.98^f^ ± 0.13	10.06^a^ ± 0.23	8.14^c^ ± 0.17	6.55^e^ ± 0.24	6.67^e^ ± 0.10	7.58^d^ ± 0.15	5.81^f^ ± 0.15	8.47^c^ ± 0.25	9.02^b^ ± 0.36
Starch (%)^o^	90.80^a^ ± 2.88	87.36^a^ ± 2.03	84.39^ab^ ± 0.89	64.64^bc^ ± 1.13	83.13^ab^ ± 1.69	30.47^cd^ ± 1.48	25.18^de^ ± 1.55	23.84^e^ ± 1.00	29.69^de^ ± 2.25	27.58^cde^ ± 1.48
Amylopectin (%)^o^	83.70^ab^ ± 2.70	77.02^a^ ± 01.85	78.74^ab^ ± 0.61	55.17^c^ ± 0.91	72.45^b^ ± 1.55	27.34^cd^ ± 1.26	22.13^e^ ± 0.51	21.45^de^ ± 0.91	26.87^cde^ ± 2.07	24.99^de^ ± 1.26
Amylose (%)^o^	7.10^d^ ± 0.18	10.34^b^ ± 0.17	5.65^e^ ± 0.27	9.47^c^ ± 0.22	10.67^a^ ± 0.14	3.13^ef^ ± 0.22	3.04^ef^ ± 1.04	2.39^g^ ± 0.09	2.81^fg^ ± 0.18	2.59^fg^ ± 0.22
**The content of hydrolyzed amino acids in rice (mg/g)**										
Total content	42.28^f^ ± 0.07	47.33^e^ ± 0.11	56.28^a^ ± 0.07	53.53^a^ ± 0.54	52.25^c^ ± 0.82	51.9^c^ ± 0.19	54.49^b^ ± 0.17	49.97^d^ ± 0.50	52.28^c^ ± 0.10	56.73^a^ ± 0.21
Aspartic acid	4.40^c^ ± 0.14	4.48^c^ ± 0.02	5.25^a^ ± 0.08	5.15^ab^ ± 0.25	5.01^ab^ ± 0.17	5.06^ab^ ± 0.25	5.23^a^ ± 0.07	4.88^b^ ± 0.22	5.01^ab^ ± 0.13	5.17^ab^ ± 0.17
Threonine	1.50^c^ ± 0.03	2.26^b^ ± 0.05	2.46^ab^ ± 0.13	2.62^a^ ± 0.07	2.31^b^ ± 0.25	2.17^b^ ± 0.31	2.31^b^ ± 0.12	2.23^b^ ± 0.10	1.68^c^ ± 0.11	2.46^ab^ ± 0.11
Serine	2.52^d^ ± 0.06	2.65^cd^ ± 0.05	3.10^ab^ ± 0.30	3.11^a^ ± 0.35	2.96^abc^ ± 0.10	2.97^abc^ ± 0.22	3.03^abc^ ± 0.24	2.72^bcd^ ± 0.21	2.81^abcd^ ± 0.13	3.15^a^ ± 0.10
Glutamic acid	8.89^d^ ± 0.15	9.47^cd^ ± 0.04	11.29^a^ ± 0.03	11.45^a^ ± 0.31	10.60^b^ ± 0.63	10.42^b^ ± 0.20	10.96^ab^ ± 0.66	9.80^c^ ± 0.11	10.62^abcd^ ± 0.26	11.29^a^ ± 0.11
Glycine	2.34^a^ ± 0.12	2.31^a^ ± 0.16	2.67^a^ ± 0.16	2.66^a^ ± 0.40	2.51^a^ ± 0.10	2.54^a^ ± 0.26	2.62^a^ ± 0.05	2.39^a^ ± 0.24	2.54^a^ ± 0.13	2.67^a^ ± 0.12
Alanine	2.83^c^ ± 0.19	2.91^bc^ ± 0.67	3.49^a^ ± 0.10	3.51^a^ ± 0.33	3.11^abc^ ± 0.23	3.15^abc^ ± 0.20	3.28^abc^ ± 0.15	2.97^abc^ ± 0.24	3.22^abc^ ± 0.12	3.42^ab^ ± 0.20
Cysteine	0.16^b^ ± 0.09	0.00^c^ ± 0.00	0.22^ab^ ± 0.07	0.20^ab^ ± 0.04	0.00^c^ ± 0.00	0.20^ab^ ± 0.06	0.18^ab^ ± 0.06	0.21^ab^ ± 0.03	0.16^b^ ± 0.10	0.30^a^ ± 0.07
Valine	2.85^c^ ± 0.22	2.82^c^ ± 0.12	3.51^a^ ± 0.20	3.51^a^ ± 0.16	3.03^bc^ ± 0.17	3.15^abc^ ± 0.18	3.34^ab^ ± 0.11	2.97^bc^ ± 0.47	3.19^abc^ ± 0.04	3.35^ab^ ± 0.11
Methionine^o^	0.00^b^ ± 0.00	0.00^b^ ± 0.00	0.00^b^ ± 0.00	0.00^b^ ± 0.00	0.00^b^ ± 0.00	0.00^b^ ± 0.00	0.00^b^ ± 0.00	0.85^a^ ± 0.15	1.07^a^ ± 0.13	0.87^ab^ ± 0.28
Isoleucine	1.82^b^ ± 0.03	1.95^ab^ ± 0.19	2.31^a^ ± 0.02	2.33^a^ ± 0.0.04	2.10^ab^ ± 0.18	2.08^ab^ ± 0.56	2.22^ab^ ± 0.08	1.96^ab^ ± 0.08	2.15^ab^ ± 0.08	2.23^ab^ ± 0.31
Leucine	3.95^d^ ± 0.40	4.23^cd^ ± 0.11	5.04^a^ ± 0.24	4.99^ab^ ± 0.33	4.64^abc^ ± 0.08	4.58^bc^ ± 0.26	4.85^ab^ ± 0.13	4.28^cd^ ± 0.07	4.74^ab^ ± 0.15	5.02^ab^ ± 0.27
Tyrosine	1.74^a^ ± 0.14	1.88^a^ ± 0.10	2.34^ab^ ± 0.07	2.25^ab^ ± 0.65	2.17^ab^ ± 0.18	2.10^a^ ± 0.20	2.25^ab^ ± 0.13	1.98^a^ ± 0.18	2.13^ab^ ± 0.21	2.35^b^ ± 0.26
Phenylalanine	2.45^b^ ± 0.08	2.72^ab^ ± 0.27	3.34^a^ ± 0.09	3.34^a^ ± 0.29	3.01^ab^ ± 0.71	2.96^ab^ ± 0.59	3.17^a^ ± 0.05	2.73^ab^ ± 0.49	2.95^ab^ ± 0.13	3.19^a^ ± 0.03
Histidine	1.26^a^ ± 0.07	1.19^a^ ± 0.21	1.46^a^ ± 0.00	1.45^a^ ± 0.19	1.32^a^ ± 0.17	1.33^a^ ± 0.06	1.38^a^ ± 0.25	1.21^a^ ± 0.13	1.29^a^ ± 0.03	1.39^a^ ± 0.12
Lysine^o^	1.70^b^ ± 0.19	2.55^a^ ± 0.09	2.83^a^ ± 0.03	2.29^a^ ± 0.26	2.74^a^ ± 0.20	2.74^a^ ± 0.24	2.78^a^ ± 0.19	2.64^a^ ± 0.16	2.08^ac^ ± 0.18	2.78^a^ ± 0.18
Arginine	3.75^d^ ± 0.05	3.76^d^ ± 0.08	4.45^cd^ ± 0.02	4.42^ab^c ± 0.13	4.39^abc^ ± 0.20	4.16^bcd^ ± 0.57	4.53^ab^ ± 0.23	4.05^cd^ ± 0.23	4.31^abc^ ± 0.13	4.62^a^ ± 0.11
Proline	2.13^b^ ± 0.15	2.15^b^ ± 0.08	2.53^ab^ ± 0.05	2.63^a^ ± 0.33	2.35^ab^ ± 0.14	2.29^ab^ ± 0.39	2.38^ab^ ± 0.13	2.11^b^ ± 0.15	2.36^ab^ ± 0.12	2.50^ab^ ± 0.42
**The content of higher alcohols in rice wine (mg/L)**										
Total content^o^	406.03^c^ ± 3.33	487.45^a^ ± 18.04	437.95^b^ ± 9.49	451.30^b^ ± 12.75	390.92^d^ ± 5.70	340.67^d^ ± 5.56	328.24^d^ ± 7.82	327.45^d^ ± 15.23	340.7^d^ ± 7.69	344.16^d^ ± 11.98
N-propanol	37.31^d^ ± 1.55	53.34^a^ ± 3.62	45.60^bc^ ± 1.00	50.36^ab^ ± 1.79	37.43^d^ ± 1.60	45.46^bc^ ± 4.83	45.07^bc^ ± 5.04	46.02^bc^ ± 4.14	41.78^cd^ ± 1.65	41.76^cd^ ± 4.12
Isobutanol^o^	140.69^c^ ± 4.73	210.90^a^ ± 10.58	147.41^c^ ± 6.07	167.40^b^ ± 4.13	125.25^d^ ± 3.54	102.01^e^ ± 2.01	91.28 ^e^ ± 4.96	94.72^e^ ± 4.25	90.05^e^ ± 2.54	89.67 ^e^ ± 4.60
Isoamyl alcohol	181.47^bc^ ± 1.34	177.60^c^ ± 6.07	200.53^a^ ± 4.65	190.72^b^ ± 7.48	177.95^c^ ± 3.54	152.16^de^ ± 1.77	149.82^de^ ± 5.45	146.44^e^ ± 10.30	155.30^de^ ± 4.56	159.02^d^ ± 3.62
Phenylethanol	46.56^b^ ± 1.68	45.61^bc^ ± 0.34	44.41^bcd^ ± 1.79	42.82^cde^ ± 1.75	50.30^a^ ± 0.56	41.04^de^ ± 0.04	42.07^de^ ± 1.85	40.28^e^ ± 4.83	53.57^a^ ± 0.52	53.71^a^ ± 0.24
**The content of ethanol in rice wine (% vol)**										
Middle point of fermentation^o^	13.97^a^ ± 0.58	11.33^cd^ ± 1.67	13.3^ab^ ± 0.00	12.3^bcd^ ± 0.00	12.3^cd^ ± 0.00	12.3^bcd^ ± 0.00	11.4^d^ ± 0.00	12.97^abc^ ± 0.58	12.63^abcd^ ± 0.58	12^cd^ ± 0.52
End point of fermentation^o^	19^a^ ± 0.00	18.7^ab^ ± 0.52	18.1^abc^ ± 0.00	17.1^d^ ± 0.00	17.1^d^ ± 0.00	17.1^d^ ± 0.00	17.1^cd^ ± 0.00	18.1^ab^ ± 0.00	17.77^bcd^ ± 0.58	18.7^ab^ ± 0.52

* The cultivar name is unknown.

o The significance was obtained using non-parametric test. The lowercase letters (a, b, c, d, e, f, g) represent the significant difference among different rice cultivars, which was analyzed by one-way ANOVA or non-parametric test (*P* < 0.05).

### The detection of protein, starch, and hydrolyzed amino acids in rice

The protein content in different rice cultivars was detected by the biuret method using bovine serum protein to build the standard curve between protein content and absorbance value (Sun and Hou, 2005, *R*^2^ = 0.9922). The rice was smashed and filtered through a 100-mesh sieve, and 0.5 g of the rice flour was mixed with 12 mL of biuret reagent and diluted into 15 mL of solution. After 30 min, the absorbance value of the prepared solution was obtained by measuring at 550 nm with a Varioskan Flash microplate reader (Thermo Fisher Scientific, USA).

The starch content in different rice was analyzed by the double-wavelength method using amylopectin and amylose to build the standard curve between starch content and absorbance value (He et al., 2022). And a good linear relationship was obtained for amylopectin (*R*^2^ = 0.9987) and amylose (*R*^2^ = 0.9900). The rice was smashed and filtered through a 100-mesh sieve, and water and fat in the rice flour were removed, respectively, by desiccation (GB5009.3-2016, 2016) and the Soxhlet extraction method (GB5009.6-2016, 2016). 0.5 g of the water-free and fat-free rice flour was mixed with 10 mL of 0.5 M KOH solution, was kept in a boiling water bath for 10 min, and was diluted into 50 mL solution. One milliliter of the solution was added to 5 mL of distilled water, adjusted to pH 3.0 using 0.1 M HCl, and added to 0.1 mL iodine reagent. After being diluted into 10 mL and being placed for 15 min, the absorbance value was measured by a Varioskan Flash microplate reader (Thermo Fisher Scientific, USA). The content of amylopectin was determined by a wavelength of 538 nm with 755 nm as a reference wavelength. The content of amylose was determined by the wavelengths of 638 nm with 438 nm as the reference wavelength (He et al., 2022).

The content of hydrolyzed amino acids in rice was detected by an amino acid analyzer S-433D (Sykam, Germany). The rice was smashed and filtered through a 100-mesh sieve, and 0.08 g of rice flour was mixed with 5 mL of 6 M HCl. The mixture was sealed and placed under 110°C for hydrolysis, and after being cooled down, it was adjusted to pH 2 and diluted into 100 mL. After being filtered with 0.45-μm membrane, the solution was analyzed using a chromatographic column LCAK07/Li (150 mm×4.6 mm). The injection volume was 50 μL, the flow velocity of ninhydrin reagent was 0.25 mL/min, and the flow velocity of mobile phase was 0.45 mL/min. The temperature of the reactor was controlled at 130°C, and the determined wavelength for proline was 440 nm and for other amino acids was 570 nm.

### Laboratory fermentation of rice wine by *S. cerevisiae*

The laboratory fermentation of rice wine was performed using 10 different rice cultivars given in Table 1 and was prepared as Wang et al. (2020) reported. In detail, 100 g of rice was soaked in 500 mL distilled water for 10 h and then drained. The rice was sterilized at 121°C for 20 min in a 500-mL triangle flask covered with air-permeable sealing film and gauze. After cooling, 150 mL sterile water, 132.3 U α-amylase, and 3,113.6 U glucoamylase were added into the flask. The rice wine medium was mixed and maintained in a 60°C water bath for 30 min. *S. cerevisiae* FBKL2.8022 was precultured overnight in PDA broth at 28°C and was inoculated into the rice wine medium at a concentration of 1 × 10^6^ cells/mL. Fermentations were performed in sextuplicate for each rice at 30°C, and daily weight loss was recorded until the end of fermentation with no weight loss. Three fermentation points including starting point, middle point with 50% of total weight loss, and end point of fermentation were determined. The yeast population at three points were analyzed by dilution coating methods with PDA agar. In total of 1.5 mL of rice wine medium at three fermentation points was taken and centrifuged at 3,000 rpm for 5 min, and the supernatant was stored at −20°C for the detection of free amino acids and glucose. At the middle and end of fermentation, the rice wine medium was filtered with gauze, and 100 mL of filtrate was mixed with 100 mL of distilled water for distillation until collecting 100 mL of distillate. The ethanol concentration (%vol) was measured by alcoholmeter method using the distillate (GB5009.225-2016, 2016). The distillate was then sealed and stored at 4°C for the detection of higher alcohols. In addition, 1 mL of fermentation mash at the middle and end of fermentation was collected and centrifuged at 10,000 rpm for 10 min, and the pellet was added with 500 μL of RNAiso Plus and kept at −20°C for gene expression analysis. As the control panels, the 10 rice cultivars were prepared in the same way but without yeast inoculation, and the analysis of free amino acids and glucose in the control panels was performed at the same time as fermentation panels.

### Detection of free amino acids, glucose, and higher alcohols

The content of 17 free amino acids in rice medium was analyzed by an amino acid analyzer S-433D (Sykam, Germany). One milliliter of supernatant stored in 2.3 was mixed with 9 mL of 2% sulfosalicylic acid, and after 15 min, it was centrifuged at 3,000 rpm for 20 min using an Allegra X-30R centrifuge (Beckman Coulter, USA). 1.5 mL of the supernatant was analyzed after filtration with a 0.45-μm filter membrane. The determination conditions were the same as stated in 2.2. The glucose content in the rice medium was analyzed with 3,5-dinitrosalicylic acid method (He et al., 2022).

Four higher alcohols, namely, isobutanol, isoamyl alcohol, phenethyl alcohol, and n-propanol, in the distillate of rice wine were analyzed after filtration with a 0.22-μm filter membrane using GC-7890A (Agilent Appropriate Technology Co., Ltd., USA) with a DB-FFAP capillary column (30 m × 0.25 mm × 0.25 μm) and a flame ionization detector (Wang et al., 2020). The standard substances of the four higher alcohols were qualitatively analyzed by a retention time, and a quantitative analysis was conducted based on the standard curves of each higher alcohol (*R*^2^ ≥ 0.9994). The temperature procedures for gas chromatography analysis were 45°C for 3 min, heating to 120°C at the rate of 16 °C/ min, maintaining the temperature for 3 min, heating up to 220°C at the rate of 50°C/min, and holding the temperature for 5 min. The detector was maintained at 260°C. The flow rates of air were set as 300 mL/min with hydrogen and bypass being 30 and 40 mL/min separately. The injection volume of the sample was 1 μL with a split ratio of 40:1.

### Gene expression analysis

The gene expression in fermentations using five rice cultivars, namely, XHC, WC, PJ, YS, and BS, was performed by RNA extraction, cDNA synthesis, and qPCR analysis. RNA was extracted using RNAiso Plus and was purified by RNase-free recombinant DNase I as described by Wang et al. (2018). After the purity examination by electrophoresis and the concentration analysis using a NanoDrop 1,000 spectrophotometer (Thermo Fisher Scientific, USA), the RNA was synthesized into cDNA using PrimeScript^TM^ II 1st-Strand cDNA Synthesis Kit. To ensure the absence of genomic DNA in RNA and the quality of cDNA, PCR using intron-containing primers of ACT1-F and ACT1-R was performed with reaction procedures and system reported previously (Mendes-Ferreira et al., 2010; Wang et al., 2018). The qPCR analysis targeted at five genes including *ADH1, SFA1, BAT1, BAT2*, and *THI3*, which were designed from sequences in GenBank by Primer 5.0 and Oligo 6.0. Specificity of primers was checked by Primer-BLAST. *PDA1* was analyzed as a reference gene for normalization in data processing. Details of the primers used for qPCR analysis are given in Table 2. The qPCR analysis was performed using the reaction procedures as described by Wang et al. (2018). The 13-μL qPCR reaction volume contained 1 μL of the 5-fold diluted cDNA, 6 μL of SsoAdvanced Universal SYBR Green Supermix, 0.4 μM of the primers, and ddH_2_O. Each sample was analyzed in triplicate. Relative gene expression levels were calculated using the 2^−ΔΔCT^ method (Schmittgen and Livak, 2008) and were shown with the fold change of gene expression level at the middle and end of fermentation.

**Table 2:** The details of primers used for RT-qPCR analysis.

**Target gene**	**Primer name**	**Sequences (5' to 3')**	**Gene function**
ADH1	ADH1-F	GCCAGTTAAGCTACCATTA	Encoding alcohol dehydrogenase
	ADH1-R	AAGTCAGCGTGAGGACAG	
SFA1	SFA1-F	ATTGCTGCTGTTGCGTAT	Encoding bifunctional alcohol dehydrogenase /S-(hydroxymethyl) glutathione dehydrogenase
	SFA1-R	ATCGCCTACAGATTCTACGA	
BAT1	BAT1-F	CTCCGAGGCTCTTCTTTA	Encoding branched chain amino acid transaminase
	BAT1-R	GCATAGTTAGCACCCAAT	
BAT2	BAT2-F	ATGTCATTTGCTGCCCTGTG	Encoding branched chain amino acid transaminase
	BAT2-R	GCATTCATGGTGCCGACT	
THI3	THI3-F	GGTAAGGGTACAGTAAACG	Encoding ranched-chain-2-oxoacid decarboxylase
	THI3-R	GTCAGGATATGTGGCATT	
ACT1	ACT1-F*	GGATCTTCTACTACATCAGC	Encoding structural protein in cytoskeleton
	ACT1-R*	CACATACCAGAACCGTTATC	
PDA1	PDA1-F*	AATTAGCTGATGCTGCTCC	Encoding E1 alpha subunit of the pyruvate dehydrogenase (PDH) complex
	PDA1-R*	TCCCTAGAGGCAAAACCTTG	

* Primers designed by Mendes-Ferreira et al. (2010).

### Data analysis

Significance analysis and correlation analysis were performed using IBM SPSS statistics 19. In detail, significance analysis used one-way ANOVA and non-parametric test with rice name as factors and the corresponding contents of nutrient and higher alcohols as dependent variables. The homoscedasticity, normality, and independence were first analyzed before the significance analysis, and if the data owned homoscedasticity, normality, and independence, the significance analysis was performed by the one-way ANOVA. The significance level was determined by Duncan's multiple comparative analysis, and the difference was determined as significant when *P* < 0.05. If the data owned homoscedasticity and independence, but did not own normality, the significance analysis was performed by the non-parametric test. The significance level was determined by the Kruskal–Wallis test, and the difference was determined as significant when P<0.05. The significant differences were represented by lowercase letters including a, b, c, etc., with the same letter indicating no significant difference and different letters indicating significant difference. The correlation analysis used bivariate with the two-sided test and used Pearson as the correlation coefficient. The correlation was regarded as extremely strong when the coefficient ranged from 0.8 to 1.0, strong from 0.6 to 0.8, middle from 0.4 to 0.6, and weak from 0.2 to 0.4 (Wang et al., 2022). And *P* < 0.05 represented a significant correlation. The results of correlation analysis were shown by Gephi 0.9.2.

## Results

The differences among 10 rice cultivars were mainly interpreted using one-way ANOVA and non-parametric test, including differences of main nutrients in rice and rice wine and differences of higher alcohols in rice wine (Table 1). All data owned independence and homoscedasticity with the significance of median higher than 0.05. Most data owned normality with a *P*-value higher than 0.05 based on the Kolmogorov–Smirnov test, and these data were further analyzed by one-way ANOVA using Duncan's multiple comparative analysis. The data not owning normality were further analyzed by non-parametric test using the Kruskal–Wallis test to interpret the difference among 10 rice cultivars.

### The correlation of main nutrients in rice and total higher alcohols in rice wine

The protein content in rice varied from 5.81 to 10.06%. YS (long-grain non-glutinous rice from Guizhou) and YJ7 (round-grain glutinous rice from Liaoning) owned the lowest protein content, and the two long-grain non-glutinous rice cultivars from Guizhou, namely, BS and TT, owned the highest protein content. The rice with the same cultivars but from different countries (TT and WD) and harvest year (YJ7 and YJ9) had a significant difference in protein content (Table 1).

The starch content in rice varied from 23.84% to 90.80%. The three long-grain non-glutinous rice cultivars from Guizhou including BS, YS, and TT owned the highest amylopectin content (83.7, 77.02, and 78.74%, respectively), and the four round-grain glutinous rice cultivars including YJ7,YJ9, XHC, and XHY owned the lowest amylopectin content (21.45, 22.13, 26.87, and 24.99%, respectively). The non-glutinous rice from Liaoning (PJ) owned the highest amylose content (10.67%), and the three round-grain glutinous rice cultivars including YJ7, XHC, and XHY owned the lowest amylose content (2.39, 2.81, and 2.59%, respectively). The rice with the same cultivars but from different countries (TT and WD) had a significant difference in the content of amylopectin and amylose (P<0.05), while the rice with the same cultivars but from different harvest years (YJ7 and YJ9) only had a significant difference in amylose content (*P* < 0.05) as given in Table 1.

The content of hydrolyzed amino acids was also measured for better elucidating the contribution of amino acids on the formation of higher alcohols. As given in Table 1, the content of hydrolyzed amino acids ranged from 42.28 mg/g (BS) to 56.73 mg/g (XHY). The rice with the same cultivars but from different countries (TT and WD) had a similar high content of hydrolyzed amino acids with XHY. Glutamic acid (8.89– 11.45 mg/g), aspartic acid (4.40–5.25 mg/g), leucine (3.95–5.04 mg/g), and arginine (3.75– 4.62 mg/g) were the four main hydrolyzed amino acids detected in most of the 10 rice cultivars with the content of each higher than 4 mg/g. Cysteine (0.00–0.30 mg/g) and methionine (0.00–1.07 mg/g) were the two hydrolyzed amino acids with a content lower than 1 mg/g in most of the 10 rice cultivars.

The higher alcohols in rice wine made from the 10 rice cultivars ranged from 327.45 mg/L (XHY) to 487.45 mg/L (YS), with PJ and the five round-grain glutinous rice wine owning similar higher alcohol content. Isobutanol ranging from 89.67 mg/L (XHY) to 210.90 mg/L (YS) and isoamyl alcohol ranging from 146.44 mg/L (YJ7) to 200.53 mg/L (TT) were the two main higher alcohols found in rice wine. The contents of n-propanol and phenylethanol were lower ranging from 37.31 mg/L (BS) to 53.34 mg/L (YS) and 40.28 mg/L (YJ7) to 53.71 mg/L (XHY), respectively. P positive correlations were shown between the content of starch (coefficient 0.854), amylopectin (coefficient 0.839), and amylose (coefficient 0.836) in rice and the total content of four main higher alcohols in rice wine. However, the content of both protein and hydrolyzed amino acids in rice was not correlated with higher alcohols in rice wine, with correlation coefficients being 0.112, and −0.157, respectively.

### The dynamics of the content of glucose, free amino acid, and higher alcohols during rice wine fermentation process

**Figure 1:**
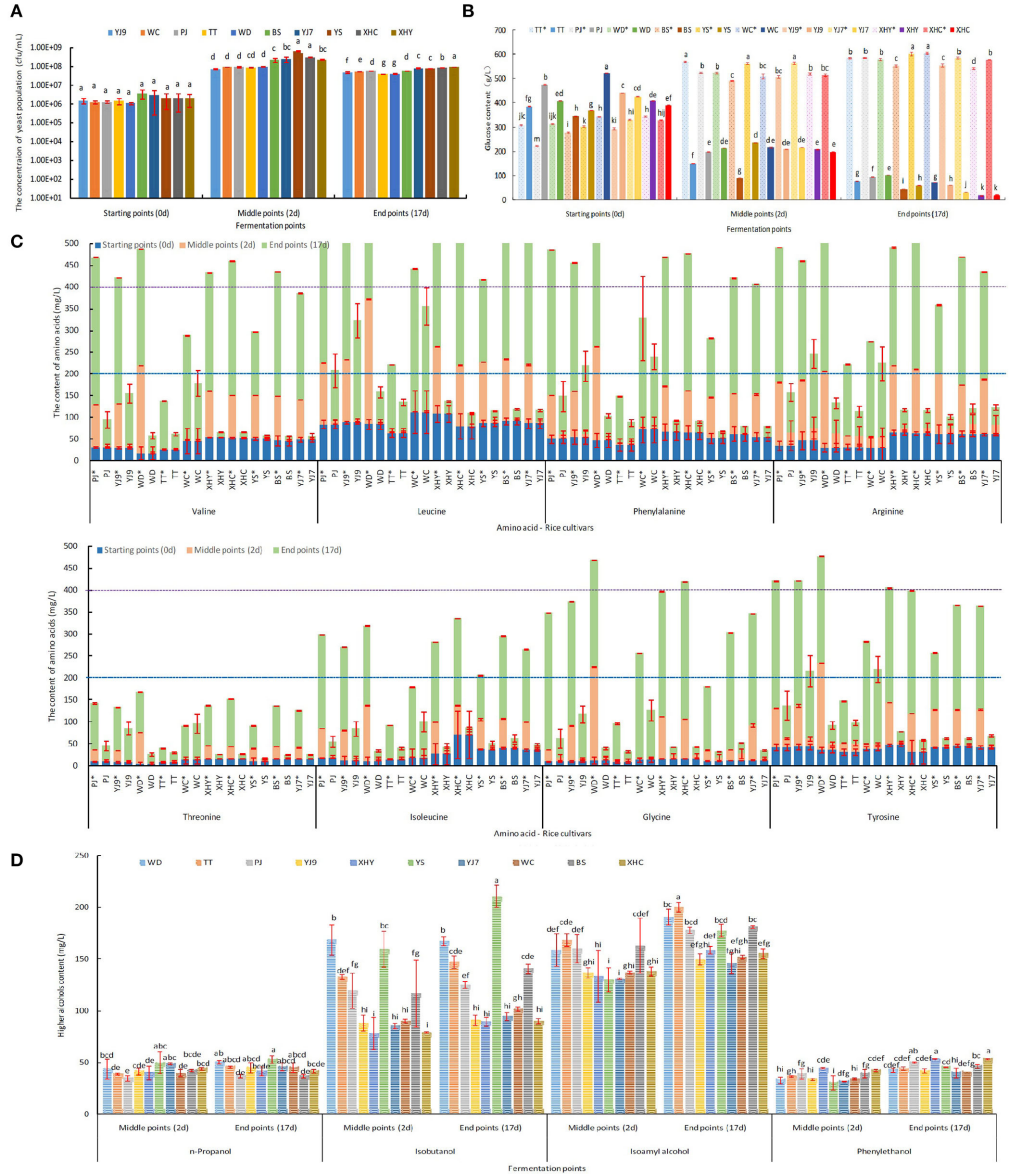
The dynamics of yeast population **(A)**, glucose content **(B)**, eight free amino acids' content **(C)**, and higher alcohols content **(D)** at different fermentation points. *Denotes the control panel without inoculation of *S. cerevisiae*. The letters (a, b, c, d, e, f, g) represent significant difference among different rice cultivars, which was analyzed by one-way ANOVA (*P* < 0.05). The significant difference in free amino acids content among rice cultivars is given in Supplementary Table 1.

*Saccharomyces cerevisiae* FBKL2.8022 could finish all rice fermentations in 17 days with culturable population concentration of 10^6^-10^8^ cfu/mL in the process (Figure 1A). *S. cerevisiae* consumed most glucose in the fermentations showing a decreasing trend of glucose content. On the contrary, the glucose content kept increasing in the control panel without yeast inoculation. Therefore, the most significant differences in glucose content between fermentation and control panels appeared at the end of fermentations with fermentation panels being lower than 100 g/L and the control panel higher than 540 g/L (*P* < 0.05). In addition, the fermentations of 10 rice cultivars also showed the differences among rice cultivars (Figure 1B). Furthermore, *S. cerevisiae* consumed the glucose fast at the beginning of rice fermentations, and all fermentations reached the middle point in 48 h (Figure 1). The fast production of ethanol verified the glucose consumption dynamic, with ethanol content reaching 11.3–13.97% vol at the middle point of fermentation in 48 h (Table 1).

Valine, leucine, phenylalanine, arginine, glycine, and tyrosine were six main free amino acids in rice mash with the sum of contents at three fermentation points higher than 400 mg/L in some rice cultivars, followed by five amino acids, namely, isoleucine, serine, glutamic acid, alanine, and lysine, with the sum of contents higher than 200 mg/L, but lower than 400 mg/L, and six amino acids with the sum of contents lower than 200 mg/L, especially for cysteine (Figure 1C and Supplementary Figure 1). The difference in free amino acids content among rice cultivars and between fermentation panels within each amino acid and fermentation point is given in Supplementary Table 1. On the whole, the content of free amino acids in the *S. cerevisiae*-inoculated fermentation panels showed a trend of first decreasing and then increasing, and the control panel showed an increasing trend in most rice cultivars (Figure 1C). When the two fermentation panels were compared at the middle and end of fermentation, all *S. cerevisiae*-inoculated fermentation panels showed a significantly lower content of free amino acids than the control panel at the middle of fermentation (*P* < 0.05). However, some *S. cerevisiae*-inoculated fermentation panels showed a significantly higher content of free amino acids than the control panel at the end of fermentation (P<0.05), including threonine in WC, aspartic acid, and glutamic acid in rice YJ9, TT, and WC, proline in seven rice cultivars except WD, XHY, and XHC, cysteine in eight rice cultivars except WD and WHY, and alanine in all the 10 rice cultivars (Figure 1C and Supplementary Figure 1). The consumption of free amino acids by *S. cerevisiae* was calculated by subtracting the content in the inoculated panel from the content in the control panel. Therefore, the content increase of the five amino acids resulted in a minus consumption of corresponding amino acids as given in Table 3. The consumption content of the sum of 17 amino acids was highest in rice wine made from BS, PJ, XHC, and XHY and was lowest in TT, WC, and YJ9. Seven amino acids showed a total consumption content higher than 200 mg/L during fermentation in some rice cultivars, including the six main free amino acids, namely, valine, leucine, phenylalanine, arginine, glycine, and tyrosine, in rice mash and serine. However, when the content of hydrolyzed amino acids in rice and the consumption contents of free amino acids was compared, it was found that more than six-fold of amino acids still remained in the rice without emission in rice wine at the end of fermentation (Table 3).

**Table 3:** The consumed and remnant amino acids in rice wine made from different cultivars.

**Amino acids**	**The consumed content**^**#**^ **/ the remnant content**^**+**^ **(mg/L)**									
	**BS**	**YS**	**TT**	**WD**	**PJ**	**WC**	**YJ9**	**YJ7**	**XHC**	**XHY**
Total content*^o/o^	2518.3^ab^ ± 11.9/ 15192.9^f^ ± 29.2	1276.3^cde^ ± 9.5/ 17655.3^ef^ ± 42.1	599.2^fb^ ± 45.5/ 21913.2^a^ ± 29.8	2148.7^bcd^ ± 80.9/ 20462.9^ab^ ± 217.9	2290.2^abc^ ± 308.0/ 18608.6^d^ ± 326.6	708.8^ef^ ± 286.1/ 20050.4^c^ ± 75.1	1208.1^def^ ± 275.5/ 20585.9^ab^ ± 68.2	2212.9^bc^ ± 22.1/ 17773.9^ef^ ± 201.2	2716.0^a^ ± 22.6/ 18194.8^e^ ± 40.8	2517.9^ab^ ± 9.8/ 20173.3^bc^ ± 84.8
Aspartic acid^o^	67.9^a^ ± 0.8/ 1692.1^e^ ± 55.4	31.8^bc^ ± 1.0/ 1758.2^e^ ± 6.7	**−7.8**^cd^ ± 5.0/ 2107.4^ab^ ± 32.0	48.5^ab^ ± 21.8/ 2011.9^bcd^ ± 98.6	28.4^abc^ ± 22.3/ 1974.4^cd^ ± 68.9	**−64.1**^d^ ± 27.1/ 2086.9^abc^ ± 100.4	**−46.6**^d^ ± 20.1/ 2139.4^a^ ± 27.0	64.9^ab^ ± 1.8/ 1886.3^d^ ± 89.4	65.6^ab^ ± 1.8/ 1939.6^d^ ± 52.9	66.9^a^ ± 0.2/ 1999.1^bcd^ ± 68.6
Threonine^o/o^	86.0^b^ ± 0.4/ 512.8^c^ ± 12.9	46.2^d^ ± 0.1/ 859.4^ab^ ± 18.6	10.0^e^ ± 2.3/ 973.6^ab^ ± 50.6	70.6^cd^ ± 3.3/ 978.2^a^ ± 26.7	71.8^cd^ ± 11.2/ 850.7^ab^ ± 100.7	−4.8^e^ ± 21.1/ 873.6^ab^ ± 123.4	23.1^e^ ± 17.9/ 901.3^ab^ ± 49.6	75.2^cd^ ± 0.9/ 816.8^ab^ ± 40.1	97.3^a^ ± 1.4/ 572.7^b^ ± 43.4	81.9^c^ ± 0.5/ 900.1^ab^ ± 44.4
Serine^o^	191.6^bc^ ± 0.1/ 816.8^f^ ± 24.0	102.2^d^ ± 1.1/ 959.4^cdef^ ± 22.6	34.1^e^ ± 3.0/ 1205.9^a^ ± 121.5	158.2^cd^ ± 5.7/ 1086.6^abc^ ± 139.9	171.7^bcd^ ± 37.1/ 1011.9^bcde^ ± 40.7	1.5^f^ ± 32.8/ 1186.1^a^ ± 87.5	53.1^e^ ± 28.0/ 1158.9^ab^ ± 96.4	173.2^bcd^ ± 1.4/ 915.6^def^ ± 84.2	220.5^a^ ± 1.4/ 901.5^ef^ ± 52.3	197.3^b^ ± 0.6/ 1063.9^abcd^ ± 38.7
Glutamic acid^o^	110.2^ab^ ± 4.9/ 3447.4^d^ ± 61.0	52.9^cd^ ± 6.7/ 3735.1^c^ ± 16.3	**−5.1**^de^ ± 8.3/ 4519.1^a^ ± 13.6	57.5^cd^ ± 13.8/ 4522.5^a^ ± 124.5	58.0^cd^ ± 32.8/ 4182.4^b^ ± 251.6	**−112.9**^e^ ± 59.6/ 4280.1^ab^ ± 81.1	**−88.6**^e^ ± 14.0/ 4471.0^a^ ± 262.3	87.4^bc^ ± 4.7/ 3833.0^c^ ± 43.5	108.9^ab^ ± 7.8/ 4138.7^b^ ± 105.4	146.8^a^ ± 8.8/ 4370.0^ab^ ± 42.1
Glycine^o^	240.4^b^ ± 1.1/ 694.0^d^47.4	131.3^ef^ ± 0.3/ 794.3^bcd^ ± 64.5	64.0^f^ ± 2.7/ 1002.8^a^ ± 65.7	220.8^d^ ± 3.1/ 841.6^bcd^ ± 158.1	268.5^ab^ ± 21.1/ 733.5^cd^ ± 40.2	131.4^ef^ ± 23.0/ 885.0^ab^ ± 104.6	181.0^e^ ± 19.7/ 865.0^abc^ ± 20.5	237.4^c^ ± 1.3/ 718.6^cd^ ± 95.2	293.4^a^ ± 1.5/ 721.8^cd^ ± 51.3	265.7^ab^ ± 0.5/ 801.13^bcd^ ± 49.0
Alanine^o^	**−87.6**^bc^ ± 2.6/ 1219.6^c^ ± 76.0	**−71.4**^ab^ ± 0.6/ 1233.8^bc^ ± 266.2	**−108.5**^cd^ ± 9.3/ 1503.7^a^ ± 41.3	**−119.34**^cd^ ± 18.69/ 1521.34^a^ ± 130.66	**−154.1**^de^ ± 33.8/ 1399.3^abc^ ± 92.0	**−250.4**^e^ ± 31.2/ 1512.0^a^ ± 80.8	**−244.6**^e^ ± 21.6/ 1556.6^a^ ± 58.2	**−58.7**^a^ ± 3.1/ 1246.3^bc^ ± 95.3	**−73.6**^ab^ ± 5.2/ 1362.0^abc^ ± 48.8	**−73.3**^ab^ ± 1.9/ 1439.7^ab^ ± 81.1
Cysteine	**−3.1**^bc^ ± 1.3/ 67.8^b^ ± 34.2	**−13.5**^de^ ± 2.1/ 13.5^c^ ± 0.0	**−9.0**^cd^ ± 0.8/ 95.0^ab^ ± 29.1	3.4^a^ ± 8.0/ 75.0^ab^ ± 14.1	**−12.0**^de^ ± 1.7/ 12.0^c^ ± 0.0	**−3.2**^bc^ ± 3.9/ 83.2^ab^ ± 25.1	**−15.6**^e^ ± 2.4/ 88.0^ab^ ± 22.5	**−15.4**^e^ ± 4.9/ 98.2^ab^ ± 12.7	**−4.2**^bc^ ± 0.2/ 68.2^b^ ± 41.4	0.0^ab^ ± 1.0/ 118.4^a^ ± 26.7
Valine^o^	276.6^ab^ ± 0.5/ 861.4^d^ ± 88.5	139.8^e^ ± 0.2/ 988.2^cd^ ± 47.7	76.0^f^ ± 3.7/ 1326.8^a^ ± 81.3	230.5^cd^ ± 7.1/ 1173.5^b^ ± 64.1	279.1^ab^ ± 19.6/ 934.5^cd^ ± 68.8	109.6^f^ ± 30.0/ 1150.0^b^ ± 73.8	170.5^d^ ± 21.2/ 1163.9^b^ ± 43.1	238.5^bcd^ ± 6.5/ 949.1^cd^ ± 186.5	295.6^a^ ± 1.3/ 978.8^cd^ ± 16.5	260.8^abc^ ± 1.0/ 1079.6^bc^ ± 43.7
Methionine^o/o^	102.3^a^ ± 2.9/ 102.3^g^ ± 0.0	56.2^bcd^ ± 0.0/ −56.2^d^ ± 0.0	24.3^d^ ± 0.6/ 24.3^a^ ± 0.0	77.7^bcd^ ± 4.5/ 77.7^e^ ± 0.0	81.6^abc^ ± 11.5/ 81.6^f^ ± 0.0	40.3^bcd^ ± 38.7/ 40.3^c^ ± 0.0	35.1^cd^ ± 4.7/ 35.1^b^ ± 0.0	75.2^bcd^ ± 1.5/ 266.4^a^ ± 60.2	80.6^bc^ ± 2.2/ 345.8^a^ ± 52.3	87.3^ab^ ± 1.9/ 258.7^a^ ± 110.3
Isoleucine^o^	170.3^ab^ ± 7.6/ 559.3^c^ ± 11.8	88.3^de^ ± 5.7/ 690.1^bc^ ± 76.0	52.0^e^ ± 2.5/ 873.6^a^ ± 6.4	159.4^b^ ± 2.6/ 771.0^ab^ ± 15.1	179.3^ab^ ± 12.7/ 622.3^bc^ ± 71.8	78.5^de^ ± 22.1/ 753.1^ab^ ± 222.7	119.4^d^ ± 16.9/ 767.8^ab^ ± 32.9	154.1^c^ ± 2.1/ 628.8^bc^ ± 30.6	186.7^a^ ± 2.2/ 674.9^bc^ ± 33.8	171.1^ab^ ± 1.8/ 719.0^abc^ ± 122.6
Leucine^o^	345.8^b^ ± 1.8/ 1232.6^f^ ± 160.4	168.5^def^ ± 0.7/ 1523.9^de^ ± 43.1	85.6^g^ ± 6.1/ 1931.2^a^ ± 97.9	287.7^cde^ ± 12.5/ 1709.1^bc^ ± 132.0	301.9^cd^ ± 38.1/ 1552.5^cde^ ± 32.4	86.9^fg^ ± 42.5/ 1746.3^b^ ± 103.3	160.2^efg^ ± 38.8/ 1781.0^ab^ ± 50.6	303.9^cd^ ± 2.2/ 1409.3^e^ ± 29.4	369.7^a^ ± 3.1/ 1525.9^de^ ± 61.6	340.4^c^ ± 2.1/ 1667.7^bcd^ ± 108.0
Tyrosine^o^	226.2^ab^ ± 1.3/ 469.4^c^ ± 56.6	115.8^cde^ ± 1.8/ 637.1^bc^ ± 41.2	48.6^f^ ± 6.3/ 888.6^a^ ± 29.1	201.9^bcd^ ± 8.4/ 697.7^ab^ ± 259.9	215.1^abc^ ± 32.9/ 654.5^bc^ ± 70.2	63.6^ef^ ± 29.5/ 777.7^ab^ ± 79.4	129.6^def^ ± 36.5/ 768.8^ab^ ± 29.6	213.3^bc^ ± 2.7/ 576.7^bc^ ± 70.9	256.8^a^ ± 2.4/ 593.2^bc^ ± 85.3	236.4^ab^ ± 0.7/ 703.2^ab^ ± 102.6
Phenylalanine^o^	249.2^bc^ ± 0.3/ 729.2^c^ ± 33.8	119.6^def^ ± 1.3/ 966.4^bc^ ± 107.8	60.9^f^ ± 7.4/ 1276.7^a^ ± 37.5	234.4^bc^ ± 5.3/ 1101.2^ab^ ± 115.4	247.1^abc^ ± 35.0/ 954.9^bc^ ± 285.4	88.5^ef^ ± 29.4/ 1096.0^ab^ ± 237.5	138.7^cde^ ± 34.7/ 1127.3^ab^ ± 21.3	230.6^bcd^ ± 0.9/ 863.0^bc^ ± 195.2	291.4^a^ ± 2.3/ 888.6^bc^ ± 52.3	271.2^ab^ ± 1.2/ 1002.8^bc^ ± 10.6
Histidine^o^	54.4^b^ ± 0.6/ 449.6^b^ ± 28.8	28.7^def^ ± 0.6/ 446.5^b^ ± 85.0	19.8^f^ ± 0.7/ 564.6^a^ ± 0.8	55.6^a^ ± 0.6/ 254.4^ab^ ± 76.2	58.6^a^ ± 1.7/ 468.6^ab^ ± 67.5	22.7^ef^ ± 10.5/ 508.5^ab^ ± 24.1	42.8^cde^ ± 5.2/ 508.4^ab^ ± 99.2	49.2^bcd^ ± 3.3/ 432.8^b^ ± 52.5	58.6^a^ ± 0.5/ 455.8^ab^ ± 13.1	52.8^bc^ ± 0.6/ 504.8^ab^ ± 49.1
Lysine^o/o^	137.1^a^ ± 0.9/ 541.3^c^ ± 77.9	64.2^cde^ ± 2.3/ 955.8^ab^ ± 34.2	15.9^e^ ± 10.4/ 1114.9^a^ ± 10.1	108.1^bc^ ± 8.8/ 1060.6^ab^ ± 102.0	110.4^abc^ ± 17.6/ 987.2^ab^ ± 80.1	24.8^e^ ± 25.6/ 1069.7^ab^ ± 96.6	52.6^de^ ± 22.1/ 1059.0^ab^ ± 76.2	104.12^bcd^ ± 0.7/ 950.6^ab^ ± 64.7	129.2^ab^ ± 6.1/ 701.6^b^ ± 73.4	108.0^bc^ ± 2.3/ 1002.4^ab^ ± 72.1
Arginine^o^	260.2^a^ ± 10.3/ 1239.0^d^ ± 19.1	130.8^def^ ± 5.6/ 1374.8^cd^ ± 34.0	108.0^ef^ ± 12.0/ 1672.4^a^ ± 8.9	230.6^abc^ ± 13.0/ 1535.8^abc^ ± 53.4	218.7^bcd^ ± 20.6/ 1538.5^abc^ ± 79.7	50.6^f^ ± 38.5/ 1613.8^ab^ ± 228.6	102.1^ef^ ± 33.3/ 1709.1^a^ ± 91.2	205.9^cde^ ± 6.4/ 1414.1^c^ ± 93.6	252.1^ab^ ± 4.4/ 1469.9^bc^ ± 52.1	230.7^bc^ ± 3.2/ 1615.3^ab^ ± 43.0
Proline^o^	**−22.8**^cd^ ± 7.1/ 876.4^ab^ ± 58.6	**−14.2**^c^ ± 2.4/ 874.2^ab^ ± 32.7	**−37.0**^**d**^^ef^ ± 4.2/ 1048.6^a^ ± 20.3	1.4^ab^ ± 4.2/ 1051.0^a^ ± 132.4	–**32.9**^**c**^^de^ ± 17.7/ 972.1^ab^ ± 57.8	**−107.1**^f^ ± 26.9/ 1021.9^ab^ ± 156.5	**−78.7**^**e**^^f^ ± 17.9/ 1029.5^ab^ ± 50.2	**−6.5**^b^ ± 0.2/ 848.9^b^ ± 60.0	9.6^a^ ± 2.3/ 933.6^ab^ ± 47.0	0.2^ab^ ± 1.9/ 1001.4^ab^ ± 169.4

* The total consumption of free amino acids during rice wine fermentation only sums up the positive consumption content of each amino acids.

# The positive value of consumed free amino acids meant the level at end of fermentation was lower than control panel without *S. cerevisiae* inoculated, while the negative value marked in bold meant that was higher than control panel.

+ The remnant content represented the total amino acids remained in rice wine and thus was calculated using the hydrolyzed amino acids in rice minus the consumed amino acids during fermentation.

o The significance was obtained using non-parametric test. The lowercase letters (a, b, c, d, e, f, g) represent significant difference among rice wine fermentations made from different cultivars, which was analyzed by one-way ANOVA or non-parametric test (*P* < 0.05).

Higher alcohols were mainly produced in the former fermentation period as shown in Figure 1D. In detail, no significant difference in n-propanol content was found between the middle and end of fermentation using the same rice cultivars (*P* < 0.05), while a significant increase of phenylethanol was found in all the 10 rice cultivars (*P* < 0.05). In addition, a significant increase of isobutanol was only found in rice YS and BS between the middle and end of fermentation (*P* < 0.05), and a significant increase of isoamyl alcohol was found in rice WD, TT, XHY, and YS (*P* < 0.05). The content of higher alcohols varied among the 10 rice cultivars, with YS showing the highest total content, n-propanol, and isobutanol, TT showing the highest content of isoamyl alcohol, and PJ, XHC, and XHY showing the highest content of phenylethanol (Table 1 and Figure 1D). The highest content of n-propanol in the middle of fermentation was detected from rice mash using YS and YJ7, with the highest isobutanol at the middle of fermentation being detected from WD and YS, isoamyl alcohol from TT and BS, and phenyl ethanol from XHY and XHC.

### Correlation analysis between the consumption of main nutrients and the content of higher alcohols during fermentation process

**Figure 2:**
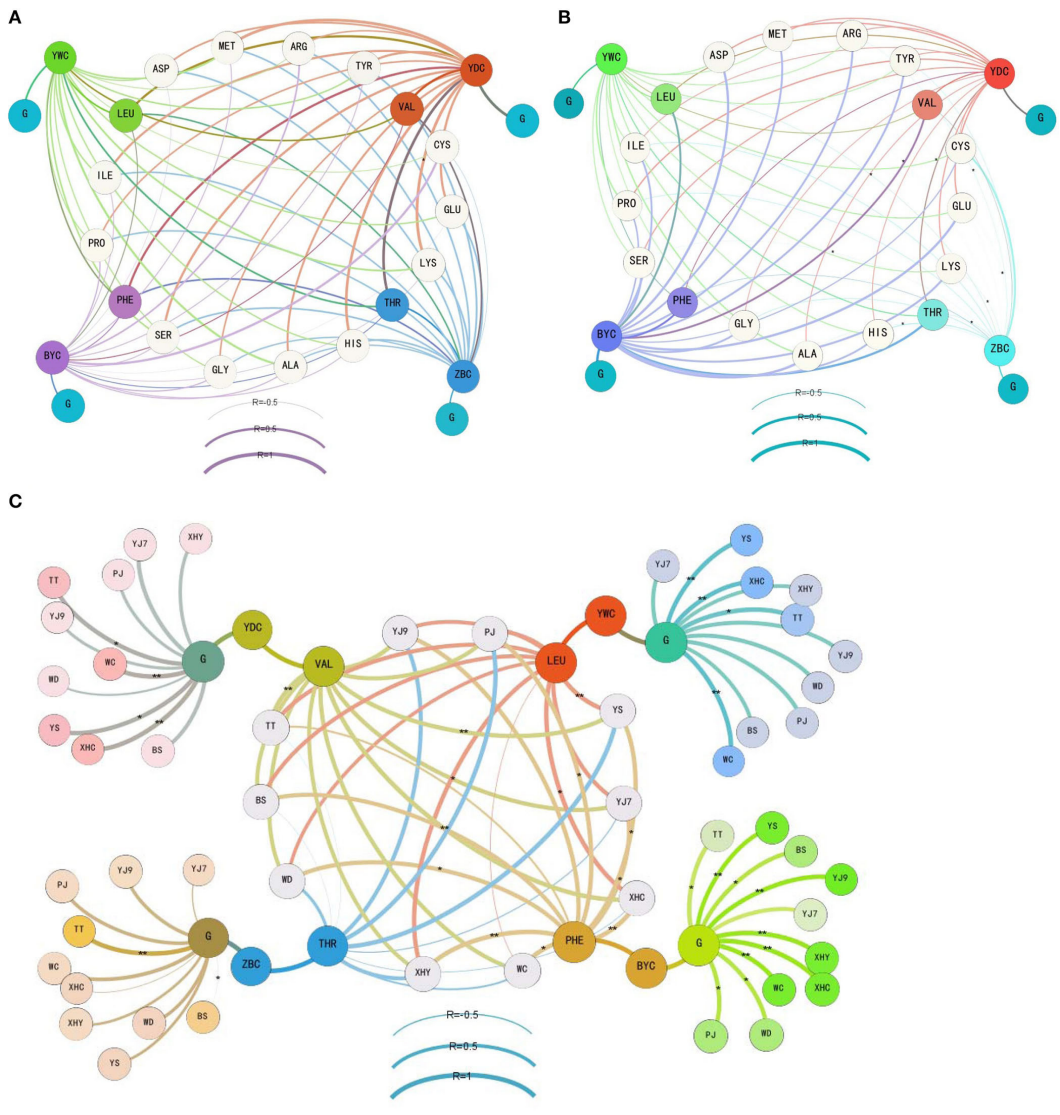
The correlation between the consumption of glucose and amino acids and the formation of four main higher alcohols during the former **(A)**, latter **(B)**, and whole **(C)** periods of the rice wine fermentations. YWC represents isoamyl alcohol, YDC represents isobutanol, BYC represents phenylethanol, ZBC represents n-propanol, G means glucose, and free amino acids are shown using the abbreviation of three letters. The details of rice cultivars are given in Table 1. *denotes a correlation with significance *P* < 0.05, and ** denotes a correlation with significance *P* < 0.01.

As the main precursors of higher alcohols, the consumption of glucose and 17 amino acids was correlated with the content of four main higher alcohols during different fermentation periods (Figure 2). The glucose consumption was strongly correlated with the formation of isobutanol (coefficient 0.629) during the former fermentation period, weakly correlated with isoamyl alcohol (coefficient 0.238), and not correlated with the other two higher alcohols (coefficient less than 0.2). The consumption of four amino acids was strongly correlated with isobutanol during the former fermentation period, including threonine (0.612), methionine (0.668), histidine (0.626), and lysine (0.7). Middle correlations were found between the consumption of serine and isobutanol (0.459), glutamic acid and isobutanol (0.458), and cysteine and phenylethanol (0.454) during the former fermentation period (Figure 2A). When the fermentation entered the latter period (Figure 2B), a weak correlation was found between glucose consumption and phenylethanol (0.334) and between five amino acids and phenylethanol including aspartic acid (0.31), threonine (0.291), serine (0.269), glutamic acid (0.313), and alanine (0.381). A middle correlation was found between proline and phenylethanol (0.461).

When the correlation analysis focused on threonine and n-propanol, valine and isobutanol, leucine and isoamyl alcohol, phenylalanine and phenylethanol, and glucose and the four higher alcohols during the whole fermentation period (Figure 2C), strong correlation only appeared in rice wine using some cultivars. In detail, a strong correlation between threonine and n-propanol was found in fermentation using YJ9 (0.729), XHY (0.662), YS (0.938), and PJ (0.875). A strong correlation between valine and isobutanol was found in all fermentations (coefficient higher than 0.8 in seven rice cultivars). A strong correlation between leucine and isoamyl alcohol was found in fermentations using eight rice cultivars except WD and WC. An extremely strong correlation was found between phenylalanine and phenylethanol in fermentations using eight rice cultivars except TT. The strong correlation was built in all fermentations between glucose and isoamyl alcohol and between glucose and phenylethanol, while it was only built in TT (0.948) and WC (0.634) between glucose and n-propanol and between glucose and isobutanol in YJ7 (0.786), TT (0.898), WC (0.973), XHC (0.960), YS (0.891), and BS (0.683).

**Figure 3:**
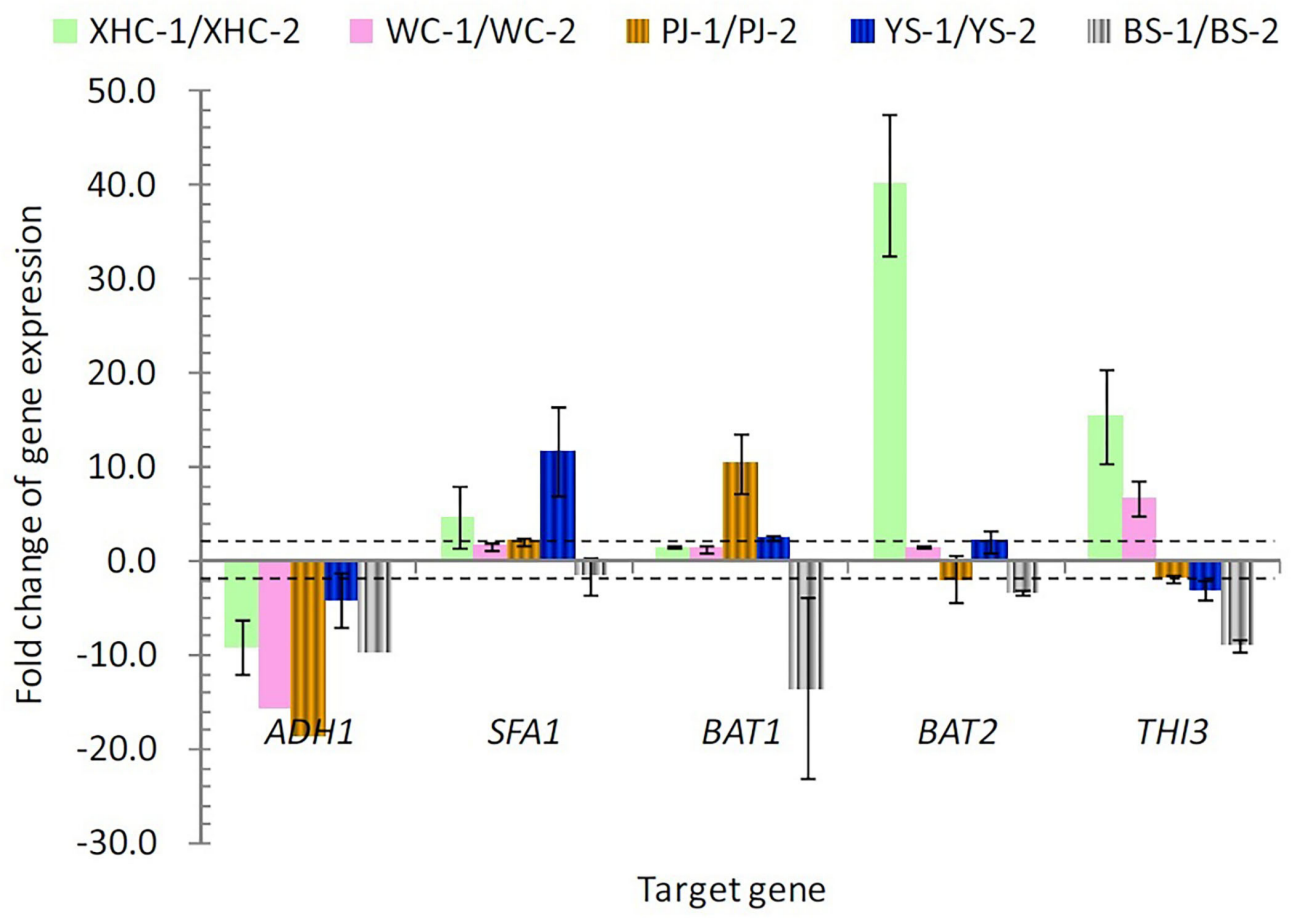
The expression change in five target genes between the middle and end of fermentation. The dotted lines denote the fold change exceeding two. 1 and 2 denote the middle and end point of fermentation.

Further gene expression analysis verified the variation caused by using different rice cultivars (Figure 3). XHC showed more than 10-fold change in the expression of *BAT2* and *THI3* at the middle of fermentation when compared with the end of fermentation. YS with the highest yield of higher alcohols showed more than 10-fold change in the expression of *SFA1*. PJ showed more than 10-fold change in the expression of *BAT1* (positive) and *ADH1* (negative change). More than 10-fold change in *ADH1* was also shown in WC, and all the five genes showed a negative fold change in BS, signifying a higher gene expression at the end of fermentation.

## Discussion

High-quality and safe food has attracted more and more attention in recent years (Luo et al., 2021; Tang et al., 2021). Traditionally speaking, glutinous rice has been preferably selected for making Chinese rice wine due to its higher amylopectin content and the consequent better gelatinization effect than non-glutinous rice (Jiao et al., 2017; Yuan et al., 2021). However, recent studies questioned whether all glutinous rice cultivars were merited to be more suitable for making rice wine than non-glutinous rice. These findings suggested that the selection of rice cultivars instead of rice type, that is, glutinous or non-glutinous, was more correlated with the quality of rice wine (Wang et al., 2014; Xie et al., 2016). Therefore, this study for the first time focused on the influence of the formation of higher alcohols during rice wine fermentation by using different rice cultivars from Guizhou and Northeast China. Northeast China and Guizhou, which is located in Southwest China, have been two main rice production regions with totally different climate and soil traits and therefore the rice used in this study representing the information of both cultivars and regions. Our previous studies have screened *S. cerevisiae* FBKL2.8022 with low yields of higher alcohols and high ethanol-producing ability from Guizhou traditional *Xiaoqu* and have found that rice wine contained four main higher alcohols including n-propanol, isobutanol, isoamyl alcohol, and phenylethanol (Wang et al., 2020). Further investigation on the formation differences of higher alcohols using the same strain but different rice cultivars in this study would be helpful in selecting a suitable rice material for making rice wine with low yields of higher alcohols.

The 10 rice cultivars showed different contents of protein and starch, especially for the content of starch, the five non-glutinous rice cultivars (BS, YS, TT, WD, and PJ) containing a significantly higher starch content (64–91%) than the other five glutinous rice cultivars (24–30%). The content of starch in five non-glutinous rice cultivars was similar to previous studies on 18 Korean rice cultivars (89%, Wang et al., 2014). The protein content of the 10 rice cultivars ranged from 5.8–10%, with some rice being lower than the 34 glutinous rice cultivars reported (8.9–12.3%, Xie et al., 2016). YJ7 and YJ9 were the same rice cultivars with YJ7 being stored for two years in the laboratory, and the protein content decreased in YJ7, which might be caused by the loss of salt-soluble globulin (Liu and Cheng, 2006). In addition, this study for the first time reported the content of hydrolyzed amino acids in rice, indicating glutamic acid, aspartic acid, leucine, and arginine being the four main amino acids in most of the 10 rice cultivars with the content of each higher than 4 mg/g.

From the perspective of total content of higher alcohols at the end of fermentation, rice wine made from five glutinous rice cultivars produced lower higher alcohols (lower than 350 mg/L), which was also lower than the reports of 18 Korean rice (Wang et al., 2014). Isoamyl alcohol and isobutanol were two main higher alcohols with a content higher than 80 mg/L, which was consistent with the report of rice wine made from 18 Korean rice (Wang et al., 2014). Nine rice wines showed one-third higher content of isoamyl alcohol than isobutanol, except the rice wine made from YS containing a higher content of isobutanol than isoamyl alcohol. Rice wine made from YS also contained the highest content of total higher alcohols, exhibiting the influence of rice selection. In addition, although glutinous rice showed a lower content of total higher alcohols, the two glutinous rice cultivars XHC and XHY contained the highest content of phenylethanol. The formation of higher alcohols was first analyzed by correlating the content of higher alcohols with the content of the main nutrients in rice. Interestingly, the rice wine made from the five glutinous rice cultivars contained a lower content of higher alcohols than the five non-glutinous rice cultivars. And the correlation analysis confirmed the strong correlation between starch content in rice and higher alcohol content in rice wine (0.854). However, both the protein and the hydrolyzed amino acids were not correlated with the content of higher alcohols.

The semi-solid-state fermentation of rice wine without the inoculation of *S. cerevisiae* showed the increase of glucose and free amino acids as times went on, which verified the incomplete release of nutrient with the existence of rice grain in the wine (He et al., 2022). Therefore, the carbon and nitrogen variation in rice wine fermentation was quite different from that in synthetic media or grape juice (Dzialo et al., 2017). The rice wine at the starting point was an environment with a higher level of glucose and free amino acids than grape juice. *S. cerevisiae* in rice wine fermentation showed a fast glucose consumption and ethanol production, and therefore, it reached the middle point in 48 h. More than 55% of glucose was consumed in the former fermentation period, with TT consuming the most (83%) of the 10 rice fermentations. The consumption of 17 free amino acids was more complex than the glucose due to the probable formation of amino acids by *S. cerevisiae* in the latter fermentation period. More than 50% of 17 free amino acids were consumed in the former fermentation period using rice WD, while fermentations using rice TT and WC consumed the least 17 free amino acids in the former fermentation period. Fermentations using the other seven rice showed different consumption proportions of the 17 free amino acids in the former fermentation period, with some amino acids higher than 50% and some others being minus. As the four main hydrolyzed amino acids, the consumption of glutamic acid (−112.88–146.8 mg/L) and aspartic acid (−64.09–67.95 mg/L) was much less than the consumption of leucine and arginine by the *S. cerevisiae* strain FBKL2.8022, showing its selective consumption of different amino acids during fermentation.

From the perspective of formation of higher alcohols during rice wine fermentation process, this study for the first time found that more than 75% of higher alcohols were formed in 48 h, that is, the former fermentation period. More than 85% of n-propanol in the 10 rice wines was formed in the former fermentation period, and more than 80% of isobutanol and isoamyl alcohol were produced in the former period except the rice wine made from YS. The production of phenylethanol was relatively slower than the other three higher alcohols, with 67% formation in the former period of YS, 77, 78, and 79% formation in the former period of WD, PJ, and XHC and more than 80% formation in the former period of the other six rice wines. The formation of higher alcohols was further analyzed by correlating the content of higher alcohols with the total consumption content of main nutrients in rice mash. The strong correlation (higher than 0.6) only appeared in valine–isobutanol, glucose–isoamyl alcohol, and glucose–phenylethanol in all the 10 rice wine fermentations. The correlation of other five amino acids (threonine–n-propanol, leucine–isoamyl alcohol, phenylalanine–phenylethanol, glucose–n-propanol, and glucose–isobutanol) varied significantly among fermentations using different rice cultivars. For example, the extremely strong correlation of phenylalanine–phenylethanol (higher than 0.8) was built in nine rice wine fermentations except in TT. The reports using hydrolysate of sorghum showed different effects of amino acids, confirming the correlation variation caused by a raw material (Kłosowski et al., 2015; Dzialo et al., 2017; Wang et al., 2021). The correlation between consumption of main nutrients and formation of higher alcohols was also checked in different fermentation stages. A strong correlation was built between glucose and isobutanol in the former period, with weak correlations of glucose–isoamyl alcohol in the former period and glucose–phenylethanol in the latter period being built. The contribution of high content of glucose on the formation of isobutanol was also mentioned in the former stage of maize mash fermentation (Kłosowski et al., 2015). This phenomenon indicated the influence of environment with high levels of glucose and amino acids on the formation of higher alcohols in *S. cerevisiae* cells during the early stage of fermentation (Dzialo et al., 2017). Previous studies widely reported the precursor amino acids for the synthesis of corresponding higher alcohols by Ehrlich pathway (Hazelwood et al., 2008; Kłosowski et al., 2015; Dzialo et al., 2017; Wang et al., 2019). However, a strong correlation in the former fermentation period was only found in four amino acids with isobutanol, and the precursor of isobutanol (valine) only showed a middle correlation (0.453). The precursors of the other three higher alcohols showed no correlation in the former fermentation period, and it was not improved when TT and WC were excluded from the analysis (Supplementary Table 2). In the latter fermentation periods, only one middle correlation (proline) and five weak correlations were built between amino acids and phenylethanol. The correlation analysis suggested the probable influence of non-precursor amino acids on the formation of higher alcohols, such as proline on phenylethanol, threonine, methionine, histidine, and lysine on isobutanol.

It is noteworthy that the correlation built basing on the whole fermentation process seems stronger than either fermentation periods, especially for glucose and the four precursor amino acids. The addition of some specific amino acids at certain fermentation points would be helpful in further explaining the influence of different rice materials on the formation of higher alcohols, as it has been performed in wine (Torrea et al., 2011; Martínez-Moreno et al., 2014). In addition, although starch content was more correlated with the formation of higher alcohols than protein, both glucose and some free amino acids could build the correlation during rice wine fermentations. And this study could not justify either glucose or some free amino acids being more correlated with the formation of higher alcohols based on the Harris pathway or Ehrlich pathway, respectively. Vidal et al. (2014) reported that the Ehrlich pathway played a main role in the biosynthesis of higher alcohols when a nitrogen source was sufficient and the Harris pathway played a main role when a nitrogen source was insufficient. However, this study did not find proof supporting this viewpoint. Due to the complexity and intricate nature of these metabolic pathways, the correlation does not always have the desired effect (Dzialo et al., 2017). It has been reported that overexpression of some genes related could increase the synthesis of certain higher alcohols such as *ADH6* and *ADH1* (Kondo et al., 2012; Shen et al., 2016; Dzialo et al., 2017). This study suggested that the different rice cultivars could also affect the gene expression of the same *S. cerevisiae* strain at the middle and end of fermentation. Further studies need to focus on the 48 h to decipher the formation of higher alcohols and to compare the metabolism difference caused by the addition of different nutrients.

## Conclusion

In summary, this study described the special formation traits of higher alcohols in rice wine fermentation, which was different from other alcoholic beverages and significantly correlated with the nutrient levels among different raw materials used. In the complex rice wine fermentation matrix, it seemed that the fast formation of higher alcohols (more than 75% in 48 h) and ethanol (more than 50% in 48 h) was quite consistent with the glucose consumption (more than 55% in 48 h), but not with the consumption of free amino acids, emphasizing the important role of glucose metabolism and the relevant Harris pathway in the formation of higher alcohols. This study also showed less higher alcohols in rice wines made from the five glutinous rice cultivars (WC, YJ9, YJ7, XHC, and XHY) with less starch content than the other five rice cultivars and therefore suggested that the selection of rice with less starch content would be helpful in controlling the formation of higher alcohols in rice wine industry. Further studies would remarkably focus on the early stage of rice wine fermentation to evaluate the influence of Harris pathway and Ehrlich pathway, which would provide theoretical references for considering the impact of high levels of glucose and amino acids on the formation of higher alcohols by *S. cerevisiae*.

## Data availability statement

The original contributions presented in the study are included in the article/Supplementary material, further inquiries can be directed to the corresponding authors.

## Author contributions

CW: conceptualization, methodology, writing—reviewing and editing, and funding. SQ: supervision and conceptualization. HZ: methodology. JT: methodology and resources. YH: methodology and investigation. GY: methodology, visualization, investigation, and writing—original draft. All authors contributed to the article and approved the submitted version.

## Funding

This work was supported by the National Natural Science Foundation of China (32060518, 32160544, and 31801523), Guizhou Province Science and Technology Project ([2022]006), High-Level Innovative Talents Training Project of Guizhou Province (QKHPTRC-GCC[2022]026-1), Guizhou Education Bureau Research Program (KY[2018]120), Guizhou University Talent Introduction Research Project ((2017)44, [2020]50), Excellent Young Scientific and Technological Talent Program ([2019]5645), and Zunyi City Innovative Talent Team Project ([2020]9).

## Conflict of interest

Author GY is director of Guizhou Maotai-flavored Liquor Group Production Co., Ltd. The remaining authors declare that the research was conducted in the absence of any commercial or financial relationships that could be construed as a potential conflict of interest.

## Publisher's note

All claims expressed in this article are solely those of the authors and do not necessarily represent those of their affiliated organizations, or those of the publisher, the editors and the reviewers. Any product that may be evaluated in this article, or claim that may be made by its manufacturer, is not guaranteed or endorsed by the publisher.
