# Supplementary material for: The formation of higher alcohols in rice wine fermentation using different rice cultivars
Source: Front Microbiol. 2022 Oct 28;13:978323. doi: 10.3389/fmicb.2022.978323 (PMC9650211; doi:10.3389/fmicb.2022.978323)
Supplement: Supplementary file 1 [file Table_1.DOCX]

Supplementary Figure 1 The content dynamics of nine free amino acids (serine, glutamic acid, alanine, lysine, aspartic acid, methionine, histidine, proline, and cysterine) at different fermentation points. *marked the control panel without inoculation of *S. cerevisiae*. The significant difference of free amino acids content among rice cultivars were shown in supplementary Table 1.
