# Supplementary material for: The formation of higher alcohols in rice wine fermentation using different rice cultivars
Source: Front Microbiol. 2022 Oct 28;13:978323. doi: 10.3389/fmicb.2022.978323 (PMC9650211; doi:10.3389/fmicb.2022.978323)
Supplement: Supplementary file 2 [file Table_2.DOCX]

Supplementary Table 1 The significant difference of free amino acids content among rice cultivars in each fermentation point.

| Rice cultivars in three fermentation points | Amino acids | | | | | | | | | | | | | | | | |
| --- | --- | --- | --- | --- | --- | --- | --- | --- | --- | --- | --- | --- | --- | --- | --- | --- | --- |
|  | ASP | THR | SER | GLU | GLY | ALA | CYS | VAL | MET | ILE | LEU | TYR | PHE | HIS | LYS | ARG | PRO |
| Fermentation point 0 | | | | | | | | | | | | | | | | | |
| PJ* | c | abcd | a | bcd | b | bcd | a | bcd | bc | b | ab | a | ab | b | abc | b | b |
| PJ | c | abcd | a | bcd | b | bcd | a | bcd | bc | b | ab | a | ab | b | abc | b | b |
| YJ9* | cde | bcd | d | cd | ab | bcd | a | bcd | bc | b | ab | a | ab | b | bc | ab | b |
| YJ9 | cde | bcd | d | cd | ab | bcd | a | bcd | bc | b | ab | a | ab | b | bc | ab | b |
| WD* | e | d | e | d | ab | d | a | d | c | b | ab | a | ab | b | ab | b | b |
| WD | e | d | e | d | ab | d | a | d | c | b | ab | a | ab | b | ab | b | b |
| TT* | de | cd | e | d | b | cd | a | cd | bc | b | b | a | b | b | c | b | b |
| TT | de | cd | e | d | b | cd | a | cd | bc | b | b | a | b | b | c | b | b |
| WC* | b | abc | d | abcd | ab | bc | a | abc | b | b | a | a | a | b | ab | b | b |
| WC | b | abc | d | abcd | ab | bc | a | abc | b | b | a | a | a | b | ab | b | b |
| XHY* | cde | bc | b | a | a | bc | a | a | bc | b | a | a | ab | a | ab | a | a |
| XHY | cde | bc | b | a | a | bc | a | a | bc | b | a | a | ab | a | ab | a | a |
| XHC* | cd | a | b | a | a | a | a | a | bc | a | ab | a | ab | a | ab | a | b |
| XHC | cd | a | b | a | a | a | a | a | bc | a | ab | a | ab | a | ab | a | b |
| YS* | de | abcd | c | ab | ab | abc | a | ab | a | ab | ab | a | ab | a | ab | a | b |
| YS | de | abcd | c | ab | ab | abc | a | ab | a | ab | ab | a | ab | a | ab | a | b |
| BS* | cde | bc | b | a | ab | abc | a | abc | bc | ab | ab | a | ab | a | a | a | b |
| BS | cde | bc | b | a | ab | abc | a | abc | bc | ab | ab | a | ab | a | a | a | b |
| YJ7* | a | bc | b | abc | ab | abc | a | ab | bc | ab | ab | a | ab | a | ab | a | b |
| YJ7 | a | bc | b | abc | ab | abc | a | ab | bc | ab | ab | a | ab | a | ab | a | b |
| Fermentation point 1 |  |  |  |  |  |  |  |  |  |  |  |  |  |  |  |  |  |
| PJ* | b | de | c | ab | cd | bc | ab | d | cd | d | e | d | de | b | d | bc | c |
| PJ | ef | h | f | e | fg | e | c | g | g | f | hi | g | h | d | f | f | cde |
| YJ9* | a | b | a | a | c | a | ab | a | g | a | a | a | a | a | ab | a | f |
| YJ9 | f | hi | fg | ef | g | ef | c | g | fg | f | hi | gh | hi | d | f | f | cde |
| WD* | b | a | a | ab | cd | ab | ab | a | a | a | b | a | b | a | a | bc | f |
| WD | f | i | fgh | ef | g | e | bc | gh | fg | f | d | hi | ij | d | f | f | def |
| TT* | d | g | e | cd | e | d | bc | f | e | e | g | f | g | c | e | e | f |
| TT | ef | hi | gh | ef | g | e | bc | gh | g | f | hij | g | ij | d | f | f | a |
| WC* | a | c | b | a | cd | abc | ab | c | bc | bc | ij | c | cd | d | d | cde | f |
| WC | ef | i | gh | ef | g | ef | c | gh | g | f | ij | ij | hi | d | f | f | c |
| XHY* | bc | c | b | d | a | f | ab | b | g | b | c | c | c | b | d | ab | bc |
| XHY | ef | i | h | f | g | ef | c | h | b | f | j | jk | j | d | f | f | ef |
| XHC* | f | i | h | ef | g | ef | c | h | g | f | ij | kl | j | d | f | f | ef |
| XHC | f | i | h | ef | g | ef | c | h | g | f | ij | kl | j | d | f | f | ef |
| YS* | cd | cd | cd | bc | d | bc | ab | d | bc | cd | e | de | f | b | cd | bc | f |
| YS | f | hi | h | f | g | ef | a | h | g | f | h | kl | j | d | f | f | ef |
| BS* | cd | ef | cd | cd | d | c | ab | c | cd | cd | e | e | f | b | bc | de | f |
| BS | f | hi | h | er | ef | e | ab | h | g | f | hij | kl | j | d | f | f | ab |
| YJ7* | a | f | d | ab | b | f | ab | e | d | d | f | de | ef | b | de | bcd | cd |
| YJ7 | ef | i | h | ef | g | ef | c | h | g | f | hij | l | j | d | f | f | ef |
| Fermentation point 2 |  |  |  |  |  |  |  |  |  |  |  |  |  |  |  |  |  |
| PJ* | b | b | a | c | a | e | h | a | a | a | a | a | a | a | a | a | cdef |
| PJ | c | f | g | ef | i | b | def | j | fg | a | i | h | fg | h | g | f | b |
| YJ9* | b | ab | ab | cd | b | e | h | c | cd | a | bc | a | abc | ab | bc | b | cde |
| YJ9 | bc | d | e | a | g | a | bcd | hi | d | a | f | f | d | g | ef | e | a |
| WD* | bc | bc | cde | ef | e | e | cdef | e | cd | a | d | cd | bc | ab | ab | a | c |
| WD | def | g | h | g | j | c | fg | k | gh | a | j | h | gh | h | g | fg | c |
| TT* | ef | fg | g | gh | h | e | g | i | ef | a | h | h | ef | f | g | e | efg |
| TT | de | gh | h | gh | j | c | abcd | k | gh | a | j | h | gh | h | g | gh | b |
| WC* | bc | d | e | f | e | e | bcde | f | c | a | e | e | c | d | d | d | fg |
| WC | a | cd | e | b | g | a | abc | gh | ef | a | f | f | d | f | e | e | a |
| XHY* | b | bc | bc | c | b | e | bcde | de | cd | a | cd | bc | abc | bc | cd | b | cdef |
| XHY | ef | hi | h | hi | j | d | bcde | l | hi | a | k | hi | h | h | h | hi | cdef |
| XHC* | b | a | a | de | a | e | efg | b | cd | a | ab | ab | ab | a | b | ab | cd |
| XHC | ef | hi | h | hi | j | d | bcde | l | hi | a | k | hi | h | h | h | hi | cdef |
| YS* | d | e | f | g | f | e | g | g | de | a | g | g | de | e | f | e | g |
| YS | f | i | h | i | j | d | a | l | i | a | k | i | h | h | h | i | defg |
| BS* | bc | bc | bcd | ef | c | e | bcde | cd | bc | a | bcd | cd | c | ab | ab | ab | cdef |
| BS | f | i | h | i | j | d | ab | l | i | a | k | i | h | h | h | hi | b |
| YJ7* | b | cd | de | ef | cd | e | h | f | c | a | e | e | c | c | cd | c | defg |
| YJ7 | ef | hi | h | hi | j | d | bcde | c | hi | a | k | hi | h | h | h | hi | cdef |

Note: *marked the control panel without inoculation of *S. cerevisiae*. The lowercase letters (a, b, c, d, e, f, g) represented significant difference among different rice cultivars, which was analyzed by one-way ANOVA (P<0.05).

Supplementary Table 2 The correlation between the consumption of free amino acids and the formation of four main higher alcohols during the former period of the rice wine fermentations, which was analyzed using eight rice except WD and TT.

| Amino acids | The correlation coefficient | | | |
| --- | --- | --- | --- | --- |
|  | n-propanol | Isobutanol | Isoamyl alcohol | Phenylethanol |
| ASP | 0.11 | -0.462 | -0.393 | -0.545 |
| **THR** | 0.269 | -0.046 | 0.003 | 0.277 |
| SER | 0.131 | -0.175 | -0.045 | 0.373 |
| GLU | 0.207 | -0.001 | -0.398 | -0.7 |
| GLY | -0.63 | -0.777* | -0.581 | 0.316 |
| ALA | 0.079 | **0.736**** | **0.527** | -0.292 |
| CYS | -0.533 | -0.852** | -0.269 | 0.326 |
| **VAL** | -0.113 | -0.045 | 0.131 | **0.555** |
| MET | 0.309 | 0.235 | **0.430** | **0.662** |
| ILE | -0.273 | -0.096 | -0.401 | -0.075 |
| **LEU** | -0.312 | -0.42 | 0.077 | **0.577** |
| TYR | **0.456** | -0.331 | -0.242 | 0.216 |
| **PHE** | 0.00 | -0.571 | -0.548 | 0.279 |
| HIS | 0.049 | 0.119 | -0.221 | 0.162 |
| LYS | **0.42** | **0.477** | 0.302 | 0.001 |
| ARG | 0.109 | -0.055 | -0.239 | **0.425** |
| PRO | 0.047 | -0.549 | -0.128 | **0.646** |

The coefficient in bold with underline meant middle (0.4 to 0.6) and strong (0.6 to 0.8) correlation. The free amino acids were shown using abbreviation of three letters. *marked a correlation with significance P<0.05, and ** marked a correlation with significance P<0.01.
